# Effects of Carpal Tunnel Syndrome on adaptation of multi-digit forces to object mass distribution for whole-hand manipulation

**DOI:** 10.1186/1743-0003-9-83

**Published:** 2012-11-21

**Authors:** Wei Zhang, Jamie A Johnston, Mark A Ross, Brandon J Coakley, Elizabeth A Gleason, Amylou C Dueck, Marco Santello

**Affiliations:** 1 School of Biological and Health Systems Engineering, Arizona State University, Tempe, AZ, 85287, USA; 2 Department of Physical Therapy, College of Staten Island, City University of New York, Staten Island, NY, 10314, USA; 3 Faculty of Kinesiology and Hotchkiss Brain Institute, University of Calgary, Calgary, AB, T2N 1N4, Canada; 4 Mayo Clinic Hospital, Phoenix, AZ, 85054, USA

**Keywords:** Sensorimotor memories, Grasping, Learning, Center of mass

## Abstract

**Background:**

Carpal tunnel syndrome (CTS) is a compression neuropathy of the median nerve that results in sensorimotor deficits in the hand. Until recently, the effects of CTS on hand function have been studied using mostly two-digit grip tasks. The purpose of this study was to investigate the coordination of multi-digit forces as a function of object center of mass (CM) during whole-hand grasping.

**Methods:**

Fourteen CTS patients and age- and gender-matched controls were instructed to grasp, lift, hold, and release a grip device with five digits for seven consecutive lifts while maintaining its vertical orientation. The object CM was changed by adding a mass at different locations at the base of the object. We measured forces and torques exerted by each digit and object kinematics and analyzed modulation of these variables to object CM at object lift onset and during object hold. Our task requires a modulation of digit forces at and after object lift onset to generate a compensatory moment to counteract the external moment caused by the added mass and to minimize object tilt.

**Results:**

We found that CTS patients learned to generate a compensatory moment and minimized object roll to the same extent as controls. However, controls fully exploited the available degrees of freedom (DoF) in coordinating their multi-digit forces to generate a compensatory moment, i.e., digit normal forces, tangential forces, and the net center of pressure on the finger side of the device at object lift onset and during object hold. In contrast, patients modulated only one of these DoFs (the net center of pressure) to object CM by modulating individual normal forces at object lift onset. During object hold, however, CTS patients were able to modulate digit tangential force distribution to object CM.

**Conclusions:**

Our findings suggest that, although CTS did not affect patients’ ability to perform our manipulation task, it interfered with the modulation of specific grasp control variables. This phenomenon might be indicative of a lower degree of flexibility of the sensorimotor system in CTS to adapt to grasp task conditions.

## Background

Carpal tunnel syndrome (CTS) is a compression neuropathy of the median nerve. Prolonged mechanical compression of the nerve can result in ischemic damage and/or changes in the myelination of the nerve, which in turn leads to slowing of axonal conduction velocity, nerve block, and in severe cases axonal loss [1,2]. CTS is characterized by a constellation of symptoms including aching and burning, tingling, numbness, weakness, and clumsiness in the affected hand. CTS also results in sensory deficits in the thumb, index, middle, and radial half of the ring finger (palmar and the most distal dorsal aspect of these digits). In severe cases, CTS can also cause motor deficits particularly in the thumb. Complications from CTS result in an average of 25 days lost from work per employee per year [3] with an average lifetime cost of $30,000 per individual in the U.S. [4]. Despite the high societal costs of CTS, there is little research done on the impact of CTS on activities of daily living such as grasping and manipulation. A few studies that have quantified the control of two-digit manipulation in CTS patients have revealed an intact ability to modulate grip forces with respect to load forces as a function of texture [5] and the use of excessive grip forces [6]. The former result may suggest a role for residual tactile sensitivity through afferent fibers spared by the median nerve compression, whereas the latter finding is reminiscent of compensatory strategies to prevent object slip elicited by anesthesia of the fingertips [7-10]. It should be noted, however, that activities of daily living often require multi-digit manipulation, e.g., grasping and lifting a bottle to pour a liquid in a glass or using tools such as a hammer. Furthermore, CTS might affect the control of two-digit grasping differently than five-digit grasping as two-digit grasping involves digits (thumb and index finger) that are both affected by CTS. As long as the two digits employed in a two-digit grasping are at collinear locations on the object, they do not generate a moment in the frontal plane, hence no object tilt, regardless of how much grip force they exert. In contrast, in a five-digit grasp excessive grip forces might have behavioral consequences. Because not all grip forces are collinear, they may cause unwanted moments and object tilt. To prevent these moments, fingertip forces have to be distributed such that the net moment on the object is zero. Being able to do so is important because often multi-digit forces have to be coordinated to prevent net moments while grasping or lifting the object to prevent it from rolling, e.g., spilling a liquid from a glass. Therefore, the execution of whole-hand grasping and manipulation might be challenged by CTS differently than in two-digit grasping. Last but not least, in whole-hand grasping not all digits have sensorimotor deficits. Specifically, for grasp tasks that involve all digits, CTS patients have to integrate sensory feedback from the CTS-affected *and* non-affected digits (part of the ring finger, little finger). These considerations motivated our recent study on the effects of CTS on modulation of multi-digit forces to object weight for whole-hand manipulation [11]. This work revealed that CTS patients are able to adapt grip force to object weight. Nevertheless, multi-digit force coordination in CTS patients differed from controls in several ways. Specifically, force modulation was more variable across trials in patients and they did not discriminate between lighter object weights. Another distinctive feature of CTS patients was their lower ability to minimize net moments on the object, thus potentially interfering with the task requirement to lift the object while preventing it from rolling. Furthermore, CTS patients consistently used excessively large digit forces across consecutive trials and, most importantly did so with both CTS-affected digits and digits with intact sensorimotor capabilities. We interpreted these findings as indicative of an interference of CTS-induced deficits in tactile sensitivity with the formation of accurate sensorimotor memories of previous manipulations. Consequently, CTS patients might have used compensatory strategies to maximize grasp stability at the expense of exerting consistently larger multi-digit forces than controls. The objective of the present study was to determine the effects of CTS on the ability to adapt multi-digit forces as a function of object center of mass (CM) for whole-hand manipulation. The rationale for extending our previous work to multi-digit force coordination as a function of object CM is that this scenario introduces different constraints relative to multi-digit force coordination to object weight. As noted above, the ability to manipulate an object (e.g., lifting an object maintaining its vertical orientation) with an asymmetric mass distribution requires changing fingertip force distributions such that the external moment of the object can be counteracted by the net moment generated by the digits. The extent to which this ability might be affected by CTS cannot be inferred from our previous work as it requires a significantly higher degree of *digit force individuation* than the uniform scaling of all fingertip forces observed when changing object weight [11]. Based on our previous work (see above [11]), we hypothesized that CTS patients *(1)* would maintain the ability to modulate both total digit normal force and tangential force distribution to object CM. However, we expected CTS patients to be less skilled than controls in coordinating digit normal and tangential forces, and therefore *(2)* use larger grip forces than controls with both CTS–affected and –unaffected digits, *(3)*, be less skilled than controls in generating a compensatory moment at object lift onset to counteract the external moment on the object, thus leading to larger object roll during lift, and *(4)* be characterized by higher trial-to-trial variability of compensatory moment than in controls. The rationale underlying expected findings *(2)*−*(4)* was that CTS-induced deficits in tactile sensitivity would interfere with both feedback-driven regulation of forces and the formation of accurate sensorimotor memories for anticipatory grasp control.

## Methods

### Ethics statement

All participants gave their written informed consent according to the declaration of Helsinki and the protocols were approved by the Institutional Review Boards at Arizona State University and Mayo Clinic Hospital.

### Subjects

Fourteen Carpal Tunnel Syndrome (CTS) patients (51 ± 2 years old, 3 males and 11 females) and fourteen age- and gender-matched healthy controls participated as subjects in the current study. The weight and height of the subjects averaged 84.8 ± 6.7 kg and 167.4 ± 3.9 cm for CTS patients, and 79.7 ± 3.6 kg and 169.7 ± 3.6 cm for controls, respectively. Patients were diagnosed with CTS by the same neurologist (Mayo Clinic Hospital, Phoenix, AZ) based on clinical symptoms and results of electrodiagnostic tests (Table 1; normative values are shown in Table 2; ulnar motor and sensory tests revealed normal values for all patients and are not shown in Table 1). The inclusion and exclusion criteria for CTS patients and control subjects have been reported in detail in our previous study [11]. Briefly, for inclusion in our study, CTS patients had to exhibit at minimum a prolonged median nerve distal sensory latency (antidromic or orthodromic, relative or absolute). Eligibility for participation in our study of control subjects included absence of CTS-like symptoms. Detailed clinical history of CTS patients and controls was carefully reviewed and we further verified eligibility for participation based on a list of exclusion criteria. ^a^ Only patients with idiopathic CTS were included in the study. All CTS patients and controls were right-handed (self-reported). Five CTS patients were tested on their left hand and nine patients were tested on their right hand. The tested hand of control subjects was matched to the hand tested in CTS patients. All participants were naïve to the purpose of the study.

**Table 1:** Participant’s descriptive information and CTS patient’s results of electrodiagnostic tests

**No.**	**CTS patients**									**Control**
	**Gender**	**Age**	**Handedness**	**Tested hand**	**Electrodiagnostic test results (abnormal values in bold)**^**1**^					**Age**
					**Median nerve study**	**Amplitude**^**2**^	**Velocity**^**3**^**(m/s)**	**Distal latency (ms)**	**F-wave latency**^**4**^**(ms)**	
1	F	48	R	L	Sensory	**11.4**	57	**2.8**		48
					Motor	10	54	**5.6**	29.7	
2	M	54	R	R	Sensory	**13.5**		**2.9**		54
					Motor	8.7	52	**6**	35.1	
3	F	57	R	R	Sensory	71.2	59	**2.5**		59
					Motor	11.5	57	4.1	26.1	
4	F	60	R	R	Sensory	**10**	62	**3.3**		60
					Motor	9.6	60	**4.8**	26.2	
5	F	56	R	L	Sensory	60.2		2.3*		56
					Motor	8.7	59	3.9		
6	F	30	R	R	Sensory	53.8	64	**2.4**		30
					Motor	11.7	59	3.4	24.1	
7	M	52	R	L	Antidromic sensory	15.2		**4.0**		54
					Motor	8.4		**4.8**		
8	F	56	R	R	Sensory	**17**	53	**5.4**		56
					Motor	8.8		**7.1**	31	
9	F	42	R	R	Sensory	**45.2**		**2.5**		40
					Motor	11.8	55	3.9		
10	F	55	R	R	Sensory	63.5	66	**2.8**		55
					Motor	8.9	52	**5**	25.9	
11	F	48	R	R	Sensory	51.1	62	**2.6**		47
					Motor	7.2	51	**5.3**	27.5	
12	F	47	R	L	Sensory	84.6	63	**2.5**		46
					Motor	10	51	3.9	27.1	
13	F	60	R	R	Sensory	**27.7**	59	**3.6**		59
					Motor	6.1	53	**5.8**	28.2	
14	M	47	R	L	Sensory	**52.4**		**2.4**		47
					Motor	9.9	54	4.2		

^1^Normative values are listed in Table 2. Sensory studies are orthodromic except patient 7, who had an antidromic median sensory study.

^2^Amplitude values for sensory studies are microvolts and motor studies are millivolts.

^3,4^ Conduction velocities and F-wave latencies were normal for all nerve studies.

**Table 2:** Normative median and ulnar nerve conduction values, Mayo Clinic Arizona EMG laboratory

**Nerve**	**Age < 60**		**Age ≥ 60**^**2**^	
**Median**	Amplitude^1^	Wrist latency (ms)	Amplitude^1^	Wrist latency (ms)
Orthodromic sensory	≥50	< 2.3	M ≥ 17.4; F ≥ 40.1	< 2.5
Antidromic sensory	≥ 15	< 3.5	M ≥ 12.2; F ≥ 15.9	< 3.7
Motor	≥ 4	< 4.5	≥ 4.5	M: < 4.4; F < 3.8
**Ulnar**				
Orthodromic sensory	≥ 15	≤2.3	M ≥ 3.4 ; F ≥ 14.4	< 2.3
Antidromic sensory	≥ 10	< 3.1	M ≥ 3.9; F ≥ 15.9	M < 3.5; F < 3.1
Motor	≥ 6	< 3.6	≥ 4.8	M: < 3.2; F < 2.9

^1^Amplitude values for sensory studies are microvolts and motor studies are millivolts.

^2^ Note that some normal values for subjects 60 years old and older are gender specific. M = male; F = female.

### Apparatus

**Figure 1:**
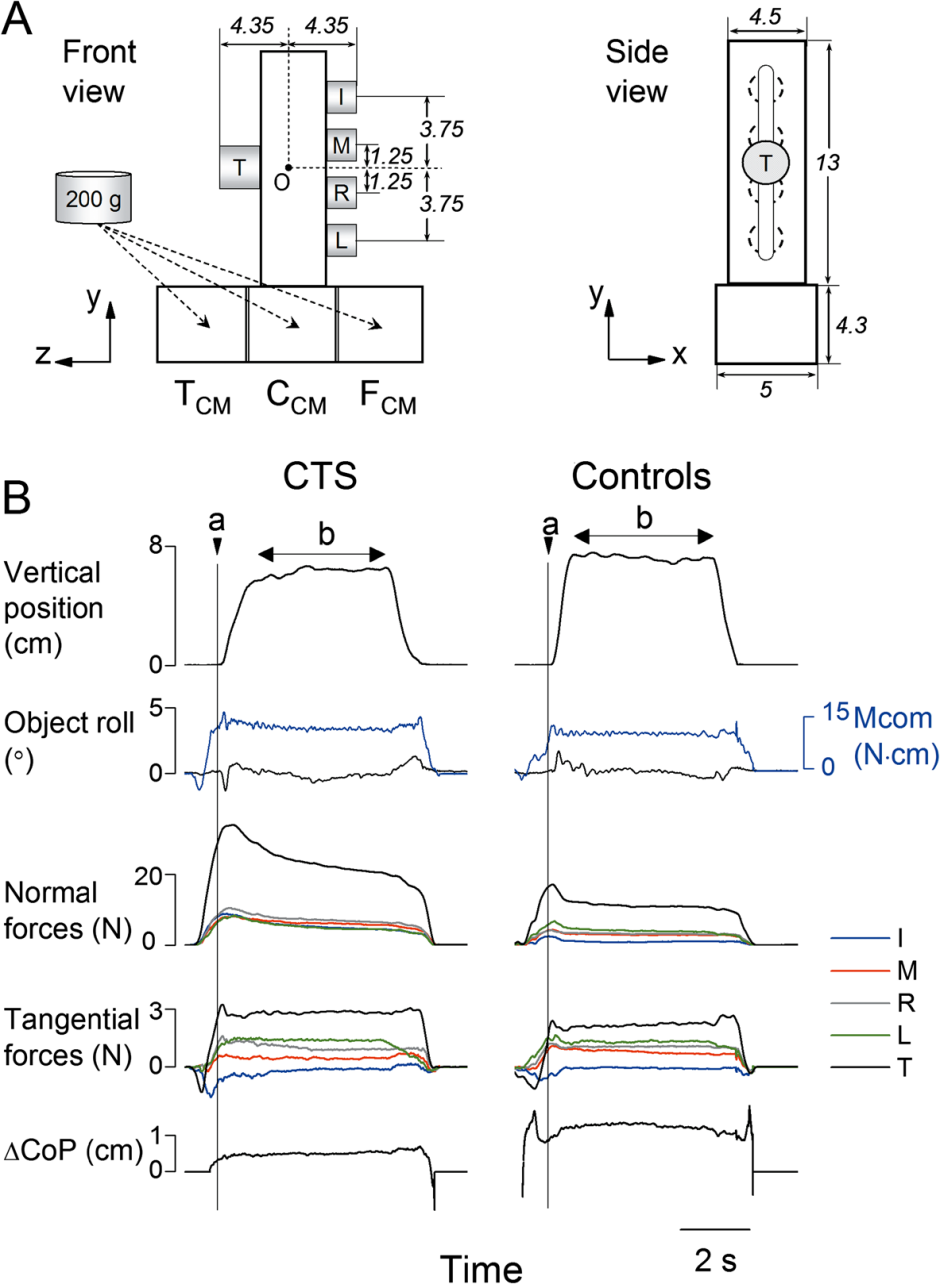
**Experimental setup and experimental variables.** Panel **A** shows the front and side views of the grip device used for the experiments and its dimensions (in cm). Force/torque sensors are mounted on both sides of the device to measure forces and moment of forces exerted by each digit (thumb, index, middle, ring, and little fingers: T, I, M, R, and L, respectively). A mass (200 g) was inserted in one of three compartments at the bottom of the grip device to change the object center of mass towards the thumb, in the center, or the finger side of the device (T_CM_, C_CM_, F_CM_, respectively). Panel **B** shows, from top to bottom, the time course of the object vertical position, object roll, compensatory moment (M*com*), individual digit normal and tangential forces (F*n* and F*tan*, respectively), and the vertical distance between center of pressure of T and the other four fingers (ΔCoP). Experimental variables are aligned with object lift onset (‘a’, vertical line). Note that analysis of digit forces during object hold (‘b’) was performed on data averaged over the last 2 seconds of the hold. Data are from one representative CTS patient (S8) and her matched control (left and right column, respectively) performing the task on the fifth trial in the T_CM_ condition.

The grip device used for our experiments (Figure 1A) has been described in a previous study [11]. Briefly, one force/torque (F/T) transducer (Nano-25, ATI Industrial Automation, Apex, NC) for the thumb (T) and four F/T transducers (Nano-17), one for each finger (I, index; M, middle; R, ring; L: little), were used to measure three force and three moment-of-force components produced by each digit. The center of the thumb sensor was aligned with the midpoint between the middle and ring finger sensors. The surface of each sensor was covered with insulating circular plates. An electromagnetic position/orientation tracking sensor (Polhemus Fastrak, Colchester, VT; 0.075 mm and 0.05° resolution) was affixed on the top of the grip device to measure the object position and angle about the vertical axis in the frontal plane, i.e., object roll. Three compartments underneath the grip device were used for positioning a 200 g load to change the object center of mass (CM) to the thumb side (T_CM_), center (C_CM_), or finger side (F_CM_) (Figure 1A). When the load was placed on the thumb, center, or finger side of the grip device, it generated an external moment of 12.4 and 0.7 N∙cm in the pronation direction, and 11.1 N∙cm in the supination direction about the origin ‘O’ (the approximate center of gravity of the grip device without load; Figure 1A). The mass of the grip device with the load was 545 g. Note the location of the load was not visible to the subjects during the experiment. Force and torque data from each sensor were acquired by five 12-bit A/D converter boards (National Instruments, Austin, TX) at a sampling frequency of 1 kHz. Collection of position data was triggered by the onset of force data acquisition and collected on a separate computer at a sampling frequency of 80 Hz. Force and position data were synchronized offline for analyses. Custom software (LabVIEW 6.1, National Instruments) was used to acquire, display and store force data.

### Experimental procedures

Before the experiment, subjects were asked to sit facing the grip device with the shoulder of the tested hand aligned with the grip device to ensure that the object could be comfortably grasped. Subjects were instructed to wait for a ‘go’ signal, after which they reached, grasped, lifted ~ 10 cm from the table, held for 4 s, and replaced the grip device on the table at a comfortable, self-selected pace. One of the experimenters visually verified that the subject contacted each sensor with the tip of a single digit. The only task requirement was to lift and hold the grip device vertically. When the mass was placed on the thumb or finger side, an external moment could cause the object to roll during the lift if not sufficiently compensated for. During the experiment, subjects were instructed to perform object grasping and lifting in three different CM conditions (T_CM_, C_CM_, and F_CM_) for 7 consecutive trials per CM condition. Thus, each subject performed a total of 21 trials. Note that subjects were unaware of the load location on the first trial in each CM condition, but were aware that the load location would remain the same within each block of 7 trials. The CM conditions were presented in a counterbalanced order across CTS patients. The order of CM presentation was matched between each CTS patient and his/her control. Subjects were given a minimum of 10-s rest period between trials and experimental conditions to prevent pain, fatigue, or worsening of the CTS symptoms. The entire experiment lasted approximately twenty minutes. None of our subjects reported any of these adverse reactions.

### Data processing

Our analysis focused on two epochs: (1) object *lift onset* and (2) object *hold*. Object lift onset (“a”, Figure 1B) was defined as the time at which the vertical position of the grip device crossed and remained above a threshold (mean + 2 SD of the signal baseline) for 200 ms. Object hold (“b”, Figure 1B) was defined as the time period between the end of object lift and the onset of object replacement on the table. The end of object lift was defined as the instant at which the absolute derivative of the object vertical position dropped less than 3% of its maximal value during object lift. The onset of the object replacement was defined as the instant at which the absolute derivative of the object vertical position increased more than 3% of its maximal value during the object downward movement. Object lift onset was used to examine anticipatory scaling of digit forces and moment to object CM based on previous manipulations, whereas object hold was used to evaluate subjects’ ability to adapt digit forces as a result of sensory feedback acquired following object lift onset. As force transients occur at the onset of object hold, digit forces were averaged over the last 2 s of the object hold. We analyzed the following variables:

1. *Digit forces.* Digit *normal force* (F*n*) is the force component perpendicular to the grip surface (Figure 1B). Grip force (F_*G*_) was defined as the sum of F*n* produced by all digits. Digit *tangential force* (F*tan*) is the vertical force component parallel to the grip surface produced by each digit to lift and hold the object against gravity (Figure 1B). The difference between F*tan* produced by the thumb and all fingers (ΔF*tan*) was used for analysis of moment of forces (see below).

2. *Moment of forces.* Moment of force (referred as ‘moment’ hereafter) was defined as moments exerted in the frontal plane (*yz* plane) about the origin ‘O’ (Figure 1A). Moment analysis was used to quantify the extent to which subjects could generate a moment *at object lift onset* in the direction opposite to the external moment caused by the additional load [12,13]. The task requirement of lifting the object vertically while preventing it from rolling is fulfilled when the moment generated on the object matches the external moment from lift throughout object hold. The moment generated by the subjects was defined as *compensatory moment* (M*com*) and was computed as the resultant moment produced by all the digits’ normal forces (normal moment; M*n*) and digits tangential forces (tangential moment; M*tan*). The magnitude and direction of M*n* and M*tan* are dependent on the F_*G*_ combined with the vertical distance between thumb and fingers center of pressures (CoPs) applied on each side of the grip device (ΔCoP), and ΔF*tan*, respectively. Hence, M*com* is a function of F_*G*_, ΔF*tan*, and ΔCoP (for details see [11,13]). The trial-to-trial variability of anticipatory moment production was quantified as standard deviation (SD) of M*com* at object lift onset and during hold (trial 4 to 7) per CM condition.

3. *Finger normal force sharing* (SF*n*) was calculated as F*n* exerted by each finger expressed as percentage of thumb F*n*. SF*n*_*j*_ was used to denote the normalized individual finger grip force (*i* = I, M, R, L). SF*n* is an important variable as it reflects the strategy used by subjects to modulate the resultant center of pressure on the finger side, hence M*com* magnitude and direction.

4. *Object roll.* The current task required subjects to minimize object roll during object lift and object hold. Thus, *peak object roll*[13,14] was used as a performance measure to further quantify the effectiveness of anticipatory grasp control.

5. *Object lift velocity.* To assess potential group differences in object lift velocity, we computed the first derivative of the object position from onset to end of lift. We analyzed *peak object lift velocity* to determine whether CTS patients lifted the object at different velocities from control subjects.

### Statistical analysis

To determine differences between CTS patients and controls in trial-to-trial adaptation of the task performance, we performed 3-way analysis of variance (ANOVA) with repeated measures on *(a)* M*com* at object lift onset and *(b)* peak object roll during lift, with *CM* (three levels: T_CM_, C_CM_, and F_CM_) and *Trial* (seven levels: 1^st^ through 7^th^ trials) as within-subject factors, and *Group* (two levels: CTS and controls) as the between-subject factor. Within the first 2–3 trials, both CTS and control subjects learned to develop M*com* to counteract the external torque, and therefore reduced peak roll. Specifically, the above statistical analyses on M*com* and peak roll revealed *1)* no trial-to-trial difference from 4^th^ to 7^th^ trial, and *2)* no significant *Group* × *Trial* interaction (these results are described in more detail in the Results section). Therefore, for the following analyses we omitted the *Trial* factor by using the mean of each experimental variable averaged across the last 4 trials for each CM (trial 4 through 7). To evaluate potential group differences in object lift velocity, we performed a 2-way ANOVA with repeated measures on peak object lift velocity with *Group* as the between-subject factor and *CM* as the within-subject factor. To determine the extent to which CTS and controls differed in multi-digit force coordination and modulation to object CM, we performed 3-way ANOVA with repeated measures on *(a)* grip force (F_*G*_), *(b)* ΔF*tan,* and *(c)* ΔCoP with *CM* and *Epoch* (two levels: object lift onset and object hold) as within-subject factors, and *Group* as the between-subject factor. To determine the existence of potential group differences in trial-to-trial variability of anticipatory moment production as a function of object CM, we performed 3-way ANOVAs with repeated measures on SD of M*com* with *CM* and *Epoch* as within-subject factors and *Group* as the between-subject factor. To quantify group differences in the adaptation of individual finger normal force sharing (SF*n*) to object CM, we performed 4-way ANOVAs with repeated measures with *CM, Epoch* and *Digit* (four levels: index, middle, ring, and little finger) as within-subject factors, and *Group* as the between-subject factor. Fisher’s z transformation was performed on SF*n* before performing statistical analysis. Mauchly’s test was used to test for sphericity. When sphericity assumption was violated, Greenhouse-Geisser correction was used as an alternative method. When appropriate, we performed post-hoc pairwise comparisons with Bonferroni adjustments. A significance level of *P* < 0.05 was used for all comparisons.

## Results

### Compensatory moment and task performance

**Figure 2:**
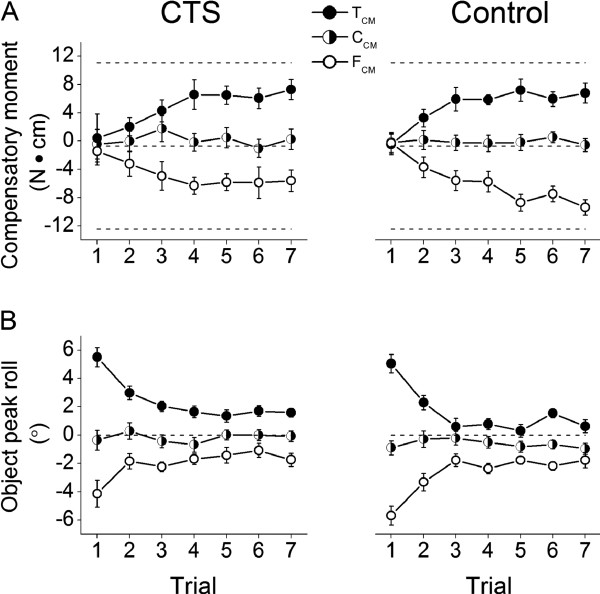
**Object peak roll and compensatory moment.** Panels **A** and **B** show trial-to-trial changes in compensatory moment at object lift onset and object peak roll during lift, respectively, for each center of mass condition and subject group. T_CM_, C_CM_, and F_CM_ denote object center of mass location on the thumb, center, and finger side of the grip device, respectively. Dashed horizontal lines in panel A denote the magnitude of the ideal moment that subjects should have generated to neutralize the external moment caused by the added mass for T_CM_, C_CM_, and F_CM_ conditions. Data are averages of all subjects. Vertical bars denote standard errors.

All subjects completed the lifting and holding task successfully without slipping or dropping the object while attempting to minimize object roll as instructed. CTS patients and controls lifted the object at similar velocities regardless of the object center of mass condition (no significant main effect of *Group*, *CM*, or interaction between these two factors*; P* > 0.05). To prevent object roll during object lift, subjects had to produce a compensatory moment cancelling the external moment before the object lift in an anticipatory fashion, i.e., at object lift onset. Figure 2A shows the compensatory moment (M*com*) produced at object lift onset and object peak roll averaged across subjects within each group from the 1^st^ through 7^th^ lift for each CM condition. As expected, subjects exerted little or no M*com* on the first lift due to lack of knowledge of object mass distribution (Figure 2A), thus resulting in a relatively large object peak roll (~5° for T_CM_, F_CM_, Figure 2B). In the subsequent lifts, however, CTS patients showed similar learning ability of M*com* production as controls (no main effect of *Group* or *Group* × *Trial* interaction on M*com* or peak object roll, *P* > 0.05 for all comparisons) as they increased M*com* in the appropriate direction required to reduce object roll (~ 2°) after the first three lifts (main effect of *Trial* on object roll: F_[6,156]_ = 52.72, *P* < 0.001; post-hoc tests showed Trial 1 > Trial 2 to 7 and Trials 2 and 3 > Trial 4 to 7) and especially so for T_CM_ and F_CM_ (main effect of *CM*; for M*com*: F_[2,52]_ = 151.451; for peak object roll: F_[2,52]_ = 23.66; < 0.001). Specifically, after experiencing the first lift both groups of subjects learned to produced supination or pronation M*com* (positive or negative values, respectively) to minimize object roll when the mass was added on the thumb or finger side (interaction effect of *Trial* × *CM*; for M*com*: F_[12,312]_ = 11.135; for peak object roll: F_[12,312]_ = 6.86; *P* < 0.001). These findings do not support one of our hypotheses that CTS would be less skilled than controls in generating M*com*. Contrary to our hypothesized group-difference in trial-to-trial variability (standard deviation) of M*com*, CTS patients did not differ significantly from controls (no main effect of *Group*; *P* > 0.05) at either object lift onset or hold.

### Components of compensatory moment

**Figure 3:**
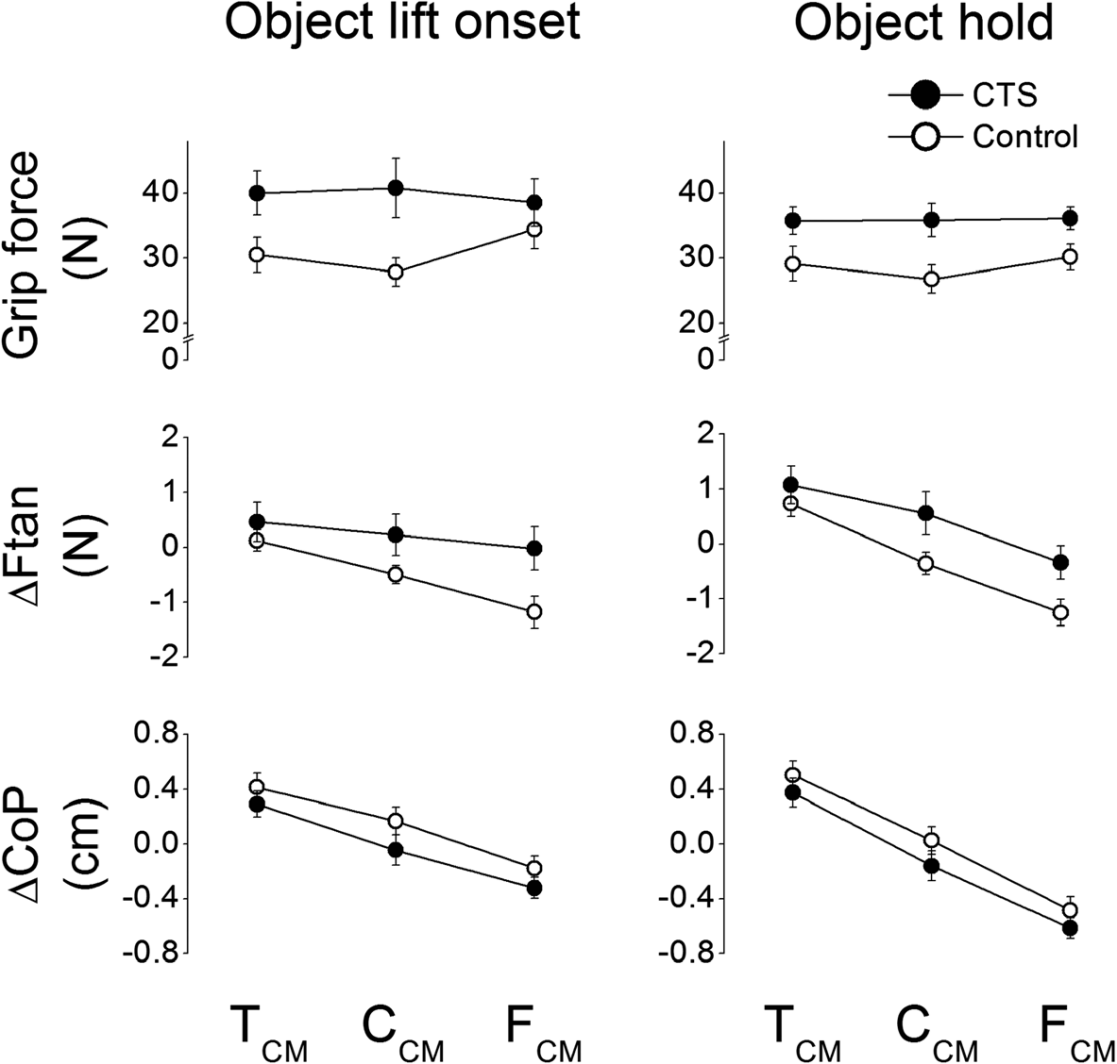
**Compensatory moment components.** Grip force (F_*G*_), the difference between thumb and finger tangential forces (ΔF*tan*), and the vertical distance between thumb and finger center of pressure applied on each side of the grip device (ΔCoP) at object lift onset and hold (left and right column, respectively). Data are mean values averaged across trial 4 through 7 for each subject group and CM condition. Vertical bars denote standard errors.

M*com* can be produced using two strategies. One strategy consists of changing the point of force application on each side of the grip device such that thumb and finger normal forces are exerted through non-collinear centers of pressure. To modulate CoP on the finger side, subjects have to change the distribution of normal forces among the fingers. A second, non-mutually exclusive strategy consists of generating asymmetrical tangential forces such that vertical forces are larger on one side of the grip device. Therefore, M*com* is dependent on the modulation of grip force, vertical distance between thumb and fingers CoPs, and the difference between thumb and the sum of the fingers tangential forces [12,13]. These variables, averaged over the last four trials of each CM condition, are shown in Figure 3 for each group and CM condition. Both groups exhibited a drop in F_*G*_ from object lift onset to object hold phase (main effect of *Epoch*: F_[1,26]_ = 4.344, *P* < 0.05). However, as expected, in both object lift onset and object hold CTS patients tended to produce larger grip force than controls for each CM condition (Figure 3, top row; main effect of *Group*: F_[1,26]_ = 5.233, *P* < 0.05). This group difference was stronger for the C_CM_ condition than lateral CM conditions (T_CM_, F_CM_; significant interaction *Group* × *CM*: F_[2,52]_ = 5.511, *P* < 0.01). Post-hoc tests further revealed that controls modulated F_*G*_ to object CM at both object lift onset and during hold, whereas CTS patients exerted similar F_*G*_ regardless of object CM in both epochs. Both groups exhibited ΔF*tan* modulation to object CM (middle row, Figure 3; main effect of *CM*: F_[2,52]_ = 50.796, *P* < 0.001; post-hoc tests revealed ΔF*tan* T_CM_ > C_CM_ > F_CM_). However, we also found a significant interaction *Group* × *CM* (F_[2,52]_ = 3.806, *P* < 0.05). Post-hoc tests revealed that CTS patients exhibited ΔF*tan* modulation (T_CM_ > C_CM_ > F_CM_) to CM during object hold but not at lift onset, whereas controls modulated ΔF*tan* in both epochs (middle row, left column, Figure 3). Unlike F_*G*_ and ΔF*tan*, the modulation of digit center of pressure (CoP) was similar in CTS patients and controls (bottom row, Figure 3; no main or interaction effect of *Group*) at both object lift onset and hold. Specifically, both groups showed ΔCoP modulation by exhibiting higher thumb CoP (positive ΔCoP) for the T_CM_ condition, collinear CoPs for the C_CM_ condition, but higher fingers’ CoP (negative ΔCoP) for the F_CM_ condition (main effect of *CM*: F_[2,52]_ = 127.285, *P* < 001). Larger separation between CoPs of thumb and fingers was observed during object hold than at object lift onset in both groups and particularly for the F_CM_ condition (bottom row, right column, Figure 3; significant interaction *Epoch* × *CM*: F_[2,52]_ = 43.825, *P* < 0.001).

### Digit normal force distribution

**Figure 4:**
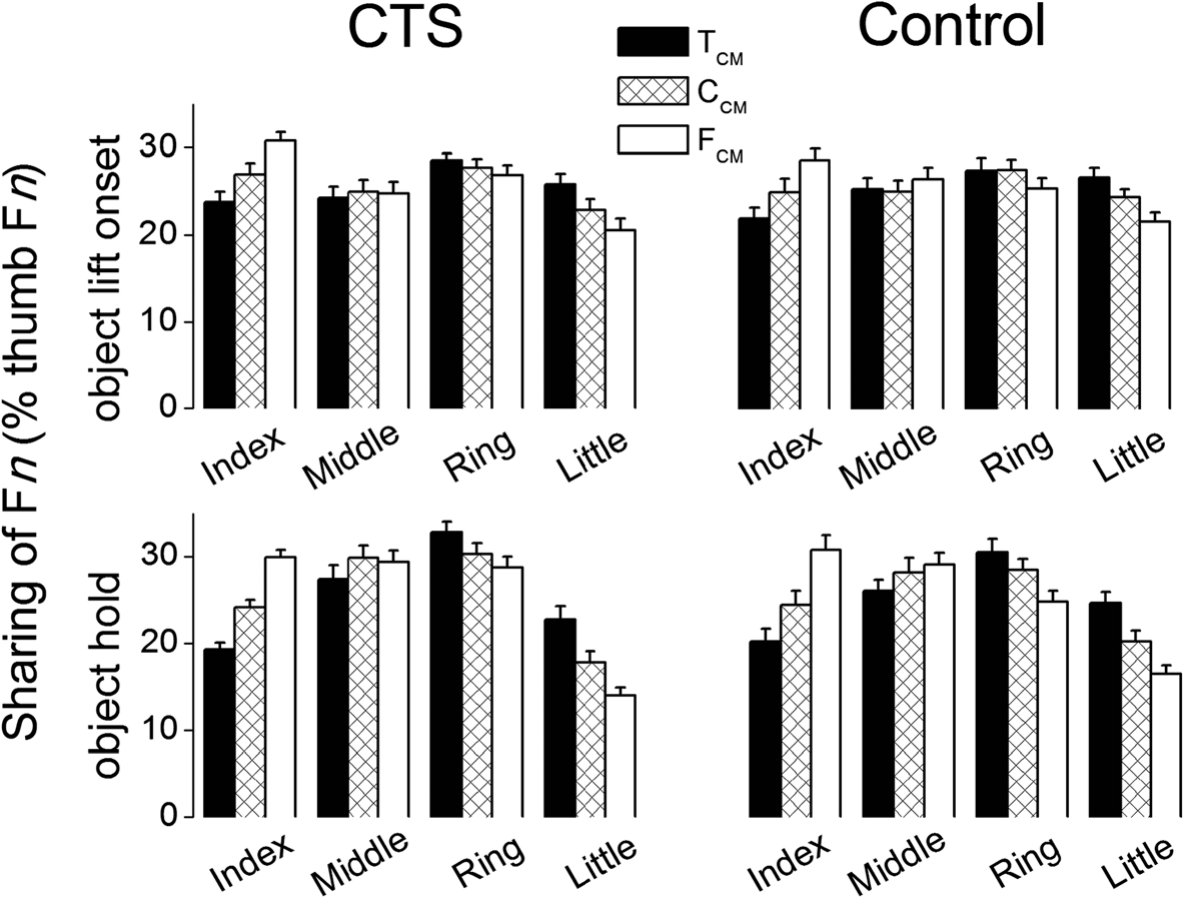
**Digit normal force sharing patterns.** Individual finger F*n* sharing expressed as percentage of thumb (T) normal force (SF*n*). SF*n* were averaged across trials 4 through 7 and across all subjects in each group and for each CM condition at object lift onset (upper panels) and hold (lower panels).

The relative relation of CoPs at thumb and fingers can be altered through 1) variation of digit initial contact position, 2) digit contact rolling along object surface, and 3) changes of normal force (F*n*) distribution among fingers. The grip device used in our current study significantly limits subjects’ use of the first two strategies, therefore the above-described modulation of digit CoP to object CM resulted primarily from changing the normal force distributions among the four fingers. Figure 4 shows the sharing patterns of finger F*n* expressed as percentage of thumb F*n* (SF*n*) averaged across the last four trials of each CM condition and subjects. At object lift onset, for the C_CM_ condition all subjects tended to evenly distribute normal forces among the fingers (range: 23.5±1.1% at little finger to 27.6±1% at ring finger). During object hold, however, most F*n* was exerted by the middle and ring fingers (~60% of thumb F*n*). For off-centered object CM conditions, both CTS and control groups modulated the distribution of finger F*n* to object CM, which was primarily reflected in the index and little fingers normal force (main effect of *Digit*: F_[3,78]_ = 10.032, *P* < 0.001; significant interaction *Digit* ×*CM*: F_[3.6,93.61]_ = 58.386, *P* < 0.001). Specifically, when object CM was changed from the thumb side (T_CM_) to the finger side (F_CM_), both groups increased and decreased the normal force exerted by the index and little finger relative to thumb normal force, respectively, throughout the object lift onset and hold. This resulted in the lowest F*n* sharing at the index and little fingers for T_CM_ and F_CM_ conditions, respectively. In contrast, neither group modulated the middle finger SF*n* as a function of object CM. The only group difference in Fn sharing patterns consisted of an epoch-dependent group difference in finger Fn distribution (significant interaction *Digit Epoch Group*: F_[3,781]_ = 3.377, *P* < 0.001). Post-hoc analysis revealed that this interaction was caused by a different pattern of re-distribution of individual finger F*n* from object lift onset to object hold in controls than in CTS patients. Specifically, CTS patients significantly increased the F*n* share at middle and ring fingers while reducing the share at index and little fingers from lift onset to object hold phase. In controls, however, such variation of individual finger share between two epochs only occurred at middle and little fingers.

## Discussion

The present study examined the effects of CTS on anticipatory control and adaptation of multi-digit forces to object center of mass in a whole-hand grasping task. CTS patients were able to satisfy the task requirement of minimizing object roll during the lift by learning to exert a compensatory moment in an anticipatory fashion to the same extent as controls. Further analysis, however, revealed that CTS patients used significantly different control strategies than healthy individuals. Specifically, patients *(a)* exerted larger grip force than controls throughout the task and regardless of object CM, and *(b)* did not modulate the magnitude of normal forces or the distribution of tangential forces to object CM in anticipatory fashion as did the controls. Nevertheless, CTS patients’ ability to modulate the *distribution* of finger normal forces, hence the finger center of pressure, to object CM effectively compensated for the lack of modulation of digit normal and tangential forces, thus resulting in successful modulation of the compensatory moment. These findings are discussed within the framework of compensatory strategies associated with sensorimotor deficits caused by CTS that selectively affect specific aspects of fine motor control.

### Multi-digit force coordination as a function of object properties in CTS

Contrary to two of our hypotheses, CTS patients learned to exert a compensatory moment at object lift onset at a similar rate and exhibited similar across-trial variability as controls (Figure 2A). By coordinating multi-digit forces to produce the required M*com*, albeit by using different strategies, both groups were able to counteract the external moment on the object, thus minimized object roll during object lift to a similar extent (Figure 2B; the relation between compensatory moment and object roll is described in our previous studies [12,13]). The observation that CTS patients can modulate M*com* to object CM is consistent with the finding of a previous study that CTS patients exhibit a residual ability to modulate multi-digit forces to object mass [11]. Although both of these tasks shared the same task requirement (lifting an object straight by minimizing object roll), in healthy individuals multi-digit force modulation to object mass is implemented through different digit force coordination patterns than modulation to object CM. Specifically, multi-digit force modulation to object mass can be attained through scaling *all digit forces uniformly* while *cancelling the net moment on the object*[15]. In contrast, digit force modulation to object CM requires *non-uniform scaling of digit forces to generate a net moment*[16,17]*.* Interestingly, in our previous study we found that CTS patients were less able to minimize the net moment when lifting an object with different symmetrical masses than were controls. In contrast, the present findings indicate no group differences in the ability to learn to generate an appropriate compensatory moment. This discrepancy indicates that learning manipulation of objects with different masses may challenge CTS patients’ ability to coordinate multi-digit forces to a greater extent than objects with different CM. Ongoing work is addressing the mechanisms underlying these differences.

### CTS patients use different multi-digit force coordination strategies from controls

Contrary to what the above findings might lead one to conclude, further analyses showed that the coordination of multi-digit forces as a function of object CM in CTS patients was significantly different from controls. Specifically, as described in the Methods, compensatory moment can be modulated through different combinations of grip force, the difference between tangential forces, and the vertical distance between thumb and finger CoP (F_*G*_, ΔF*tan*, and ΔCoP, respectively). We found that CTS patients modulated two of these three variables, F_*G*_ and ΔF*tan*, differently from controls, thus refuting our hypothesis that CTS patients would be able to modulate total digit normal and tangential force distribution to object CM.

### Grip force

As hypothesized, CTS patients exhibited significantly larger F_*G*_ than controls (Figure 3A) both before and after object lift onset. This finding was expected based on the results of our previous study of CTS patients [11] as well as the effects of reduced tactile sensitivity due to median nerve compression [6,18] or digit anesthesia [7-10]. Zhang et al. [11] suggested that exertion of excessive F_*G*_ might be a compensatory strategy to prevent object slip in response to the inability to form accurate sensorimotor memories from previous manipulations. Although this interpretation might also account for the present result, an additional and important finding here is that F_*G*_ modulation to object CM observed in controls was not found in CTS patients. Specifically, controls exerted larger F_*G*_ for the task condition that required a compensatory moment (T_CM_ and F_CM_) and smaller F_*G*_ for the C_CM_ condition where zero compensatory moment was required (Figure 3A). In contrast, CTS patients did not modulate F_*G*_ to object CM, even though in our previous study the same group of patients exhibited F_*G*_ modulation to object mass [11]. We speculate that the present lack of F_*G*_ modulation to object CM might originate from the patients’ predominant tendency of using larger than necessary F_*G*_ to prevent object slip, a tendency that was also found in our previous study. Even though this strategy is less ‘economical’ than that used by controls, CTS patients exerted the same, but larger than necessary F_*G*_ across different object CM locations to ensure attainment of one of the main task objectives: preventing object slip regardless of object mass distribution.

### Tangential force difference

Besides F_*G*_, CTS patients differed from controls also with respect to ΔF*tan* modulation to object CM. Specifically, controls learned to exert larger F*tan* on the heavier side of the object before object lift and throughout object hold (Figure 3B). This observation is consistent with studies of precision grips [12,13,19]. In contrast, CTS patients were able to learn to modulate digit F*tan* to object CM during object hold, but did not use this strategy in an anticipatory fashion, i.e., at object lift onset, despite manipulating the same object across consecutive lifts. This observation points to a feedback-driven adjustment of ΔF*tan* in CTS patients, hence to a potentially different strategy from that used for F_*G*_. This behavior is also reminiscent of the reduction in F_*G*_ from object lift onset to hold recently described in our previous study on multi-digit force modulation to object mass [11].

### Vertical distance between thumb and finger CoP

In our task, the net point of force application of the normal forces exerted by each finger is an important variable because it affects the magnitude of the compensatory moment. Specifically, the vertical distance between the (overall invariant) center of pressure of the thumb and the (variable) net center of pressure of all fingers is one of the three components, ΔCoP, whose magnitude affects the magnitude of the moment generated by F_*G*_. Using the present grip device that constrains digit placement, the primary way to modulate ΔCoP is to change the sharing of individual digit normal forces on the finger side of the device to match the normal force exerted by the thumb. At object lift onset, we found that ΔCoP was the only component of the compensatory moment that CTS modulated to object CM similarly to controls (Figure 3C). As expected from the above considerations and consistent with our previous work [11], CTS and controls exhibited similar normal force sharing patterns (Figure 4). It is therefore clear that, regardless of whether the goal is to scale all digit forces to object mass or share them in a non-uniform fashion to object CM, CTS patients are able to individuate normal forces of individual digits to the task’s mechanical requirements. According to the mechanical advantage principle, i.e., larger force production in the element(s) with longer lever arms in moment of force production [20,21], finger normal force sharing at the index and little fingers were modulated the most to object CM, this being likely due to the need modulate the net center of pressure on the finger side of the grip device as a function of object CM (Figure 3). In the present study, however, the ability to modulate normal force sharing pattern was instrumental in attaining the necessary compensatory moment through modulation of ΔCoP which, accompanied by greater F_*G*_, effectively compensated for the above-discussed lack of modulation of ΔF*tan* and F_*G*_ to object CM.

### Mechanisms underlying modulation of multi-digit forces in CTS

#### Anticipatory grasp control

Both the present and previous findings reveal that CTS patients exhibit a residual ability to learn anticipatory grasp control, as indicated by the scaling of grip force to mass [11] and compensatory moment to object CM (Figure 2A) *before* the object is lifted. The mechanisms responsible for this behavior require (1) accurate sensing of object properties, (2) the formation and retrieval of sensorimotor memories, thus leading to (3) an appropriate force output modulation (see [11] for further discussion). With regard to acquiring feedback about object properties, CTS patients are likely to rely on residual tactile sensation from the CTS-affected digits, intact sensation from digits not affected by CTS, as well as proprioceptive feedback from muscles, tendons, and joints mostly above the wrist, i.e., extrinsic finger muscles and wrist muscles. As discussed above, however, the modulation of normal and tangential forces in CTS patients was significantly different from controls, indicating that the sources of feedback spared by median nerve compression were not adequate to allow them to choose the multi-digit force coordination strategy that healthy individuals chose to implement. Specifically, unlike controls, at object lift onset patients modulated only one of the three available variables to object CM, i.e., ΔCoP. This finding can be interpreted as solving the problem of redundant degrees of freedom by ‘freezing’ some of them [22-25]. The use of this strategy might have been preferable because the modulation of one variable to CM while keeping two other variables constant (F_*G*_ and ΔF*tan*) might be easier to implement than concurrent modulation of three variables as found in controls. The selective modulation of ΔCoP also indicates that residual tactile and proprioceptive feedback (above) in CTS can be more effectively integrated with motor commands for generating individuated finger forces than for the fine scaling of finger force magnitude. This might account for patients’ reliance on exploiting finger force sharing pattern modulation to attain the desired compensatory moment.

#### Online grasp control

Following object lift and during object hold, sensory feedback about the manipulation (i.e., object tilt) becomes available through vision and residual somatosensory feedback (above), thus allowing individuals to detect errors in the anticipatory control of grasp variables. Interestingly, whereas CTS patients did not modulate ΔF*tan* to object CM at object lift onset, they did so during object hold. This suggests that the above-discussed ‘freezing of degrees of freedom’ strategy used at lift-off was deliberate, as opposed to unavoidable. Specifically, and as we suggested in our previous work [11], CTS patients might be particularly conservative before the dynamic phase of the task than during the static phase – this is because avoidance of object slip or roll during the first 100–150 ms of object lift relies on feedforward control of the compensatory moment. Following object lift, however, the predictive component of compensatory moment control can be replaced by online feedback control. In addition to somatosensory feedback from the hand and the arm, vision of object orientation might have contributed to the modulation of ΔF*tan* to object CM.

### Grasp control strategies in CTS

Our findings indicate that, to attain the required compensatory moment, controls fully took advantage of the possibility to modulate all of the three variables associated with normal and tangential force modulation as well as the vertical distance between centers of pressure. In contrast, CTS patients solved the problem of generating a given compensatory moment at object lift onset by using the same normal and tangential forces *regardless* of object CM location while modulating the finger normal force distribution. This important finding suggests that CTS patients narrow the number of available degrees of freedom or force coordination strategies to comply with the mechanical requirement of grasp tasks resulting in a reduced flexibility in the adaptability to task conditions. The extent to which these phenomena might affect CTS patients’ adaptability to manipulations that are more similar to those encountered in activities of daily living is currently under investigation.

## Endnotes

^a^Exclusion criteria for both controls and patients were: 1) clinical history or electrodiagnostic test results indicating ulnar, radial or proximal median neuropathy, brachial plexopathy, cervical radiculopathy or polyneuropathy, 2) orthopaedic, joint degeneration (i.e., arthritis, verified by x-ray) affecting the hand or cervical spine, 3) visual problems that would interfere with our grasp task, 4) co-existing central nervous system disease (e.g., multiple sclerosis, motor neuron disease, myasthenia gravis, Parkinson’s disease, dystonia) revealed in medical history 5) significant rigidity as assessed through range of motion testing, 6) active psychiatric illness, 7) pregnancy, 8) thyroid disorders, 9) introduction of clinically significant dose change of medication known to affect motor or sensory function within 3 months of enrollment, 10) history of hand surgical interventions or corticosteroid injections for carpal tunnel syndrome and/or other musculoskeletal hand disorder, and 11) older than 60 years.

## Abbreviations

CTS: Carpal tunnel syndrome, a compression neuropathy of the median nerve that results in sensorimotor deficits in the hand.; CM: Object center of mass.; T: I, M, R, L, Thumb, index finger, middle finger, ring finger and little finger, respectively.; T_CM_: Experimental condition with the object center of mass shifted to the thumb side by positioning a 200 g load at the bottom of the object.; C_CM_: Experimental condition with the object center of mass shifted to the center of the object by positioning a 200 g load at the bottom of the object.; F_CM_: Experimental condition with the object center of mass shifted to the finger side by positioning a 200 g load at the bottom of the object.; F*n*: Digit force component perpendicular to the grip surface (normal force).; F_*G*_: Grip force, defined as the sum of normal forces produced by all digits.; F*tan*: Digit force component parallel (vertical) to the grip surface (tangential force).; ΔF*tan*: The difference between tangential forces produced by thumb and all fingers.; ΔCoP: The vertical distance between center of pressure (force application point) produced by thumb and all fingers.; M*com*: Compensatory moment, defined as the resultant moment produced by all the digits with respect to the origin ‘O’ of the object in the frontal plane (Figure 1A).; M*n*: Normal moment, defined as the component of the compensatory moment resulting from all digits normal forces with respect to the origin ‘O’ of the object in the frontal plane.; M*tan*: Tangential moment, a compensatory moment component produced by all digits tangential forces with respect to the origin ‘O’ of the object in the frontal plane.; SF*n*: Finger normal force sharing pattern, defined as normal force exerted by each finger expressed as percentage of thumb normal force.

## Competing interests

The authors declare that they have no competing interests.

## Authors’ contributions

WZ, JAJ, and MS contributed to the design of the study and manuscript preparation. WZ and MS performed data processing and analyses. MR, BJC, and EG contributed to subject recruitment and screening, assisted with data acquisition, and contributed to manuscript preparation. AD contributed to the statistical design and manuscript preparation. All authors read and approved the final manuscript.
